# Capturing the transcription factor interactome in response to sub-lethal insecticide exposure

**DOI:** 10.1016/j.cris.2021.100018

**Published:** 2021-07-25

**Authors:** Victoria A Ingham, Sara Elg, Sanjay C Nagi, Frank Dondelinger

**Affiliations:** 1 Vector Biology, Liverpool School of Tropical Medicine, Pembroke Place, Liverpool, L35QA; 2 Centre for Health Informatics, Computation and Statistics, Lancaster Medical School, Lancaster University, Bailrigg, Lancaster, LA1 4YE

**Keywords:** Insecticide resistance, transcriptional response, transcription factors, Bayesian, time course, Anopheles, pesticide resistance, transcriptomics

## Abstract

The increasing levels of pesticide resistance in agricultural pests and disease vectors represents a threat to both food security and global health. As insecticide resistance intensity strengthens and spreads, the likelihood of a pest encountering a sub-lethal dose of pesticide dramatically increases. Here, we apply dynamic Bayesian networks to a transcriptome time-course generated using sub-lethal pyrethroid exposure on a highly resistant *Anopheles coluzzii* population. The model accounts for circadian rhythm and ageing effects allowing high confidence identification of transcription factors with key roles in pesticide response. The associations generated by this model show high concordance with lab-based validation and identifies 44 transcription factors putatively regulating insecticide-responsive transcripts. We identify six key regulators, with each displaying differing enrichment terms, demonstrating the complexity of pesticide response. The considerable overlap of resistance mechanisms in agricultural pests and disease vectors strongly suggests that these findings are relevant in a wide variety of pest species.

## Introduction

Insecticides are critical for control of pests in agriculture and disease vectors in public health. The intensive and widespread use of insecticides in each of these settings has led to extensive insecticide resistance (WHO 2020), which poses a threat to both food security and global health. Vector borne diseases account for more than 17% of all infectious diseases annually (WHO 2020), whilst around 35% of crops are lost to pre-harvest pests, underlining the importance of pesticide chemistries in global health and food security (Popp et al., 2013). Malaria control highlights the pivotal role of insecticides in global health with over 80% of the reductions in malaria cases since the turn of the century attributed to their use (Bhatt et al., 2015). Malaria control relies heavily on the distribution and use of insecticide treated bed nets (ITNs), which provide protection to the user and wider community protection through insecticide induced mortality of the adult *Anopheles* vectors (Hawley et al., 2003, Killeen and Smith, 2007, Killeen et al., 2011). All ITNs currently in use contain the pyrethroid class of insecticide; a fast-acting chemistry that induces immediate knockdown and mortality in susceptible mosquitoes. However, strength of resistance to pyrethroids is now such that populations of *Anopheles* can survive exposure with minimal effect on their life span (Hughes et al., 2020). Surviving sub-lethal exposures to pesticides is likely to have large and sustained consequences on the biology of the pest species.

Resistance to insecticides both in agricultural pests and disease vectors have been attributed to three characterised mechanisms; changes to the insecticide target site (Weill et al., 2004, Martinez-Torres et al., 1998), thickening of the cuticle to reduce penetrance (Balabanidou et al., 2016) and metabolic clearance through overexpression of detoxification protein families (Müller et al., 2008, Voice et al., 2015, Ingham et al., 2018). Recently, new resistance mechanisms have been reported (Ingham et al., 2018, Ingham et al., 2019) and sub-lethal exposure has been shown to induce large-scale transcriptomic changes, highlighting the complexity of the insects response to insecticides (Ingham et al., 2020).

The demonstration of large-scale changes in transcriptome post-exposure emphasises the importance of transcriptional control in response to insecticide. Despite this, the induction of genes in response to insecticides is poorly studied and the regulatory processes underlying these mechanisms have remained elusive. In most important pests, cis or trans-acting regulatory elements are yet to be identified, and little published research has focused on the role of the non-coding regulatory machinery. Although recent work has identified transcription factors involved in insecticide resistance such as two transcriptional pathways: the Nrf2-cnc pathway in both disease vectors (Ingham et al., 2017, Bottino-Rojas et al., 2018) and agricultural pests (Kalsi and Palli, 2015, Gaddelapati et al., 2018) and the ARNT-AhR in agricultural pests (Peng et al., 2017, Hu et al., 2019), no studies in either setting have examined transcriptional response in a holistic manner. The availability of transcriptomic time-series data from resistant *Anopheles coluzzii* mosquitoes post-pyrethroid exposure (Ingham et al., 2020) has provided a resource to examine the importance of multiple transcription factors in response to insecticide.

Elucidating complex gene networks from transcriptomic time course data is a fundamental problem in computational systems biology (Delgado and Gómez-Vela, 2019, Thompson et al., 2015, Jackson et al., 2020). Time course data enables measurements of mRNA levels post-perturbation and allows identification of transcripts following similar expression patters over time. Measuring changes in mRNA levels acts as a proxy for protein expression, but regulatory relationships cannot be captured by correlation alone, due to the presence of indirect regulation (gene A regulates gene B which regulates gene C), and post-transcriptional changes. To allow reconstruction of gene regulatory networks, dynamic Bayesian networks have successfully been applied to real-world time course studies (Murphy and Mian, 1999, Dondelinger et al., 2013, Dondelinger and Mukherjee, 2019), allowing identification of key regulatory pathways within a system. These models additionally allow correction for confounding factors. For example, as circadian rhythms can play a significant role in gene expression patterns over short time-scales (Rund et al., 2011), sinusoidal patterns with 24-hour period may be corrected for.

Here, we apply a modified dynamic Bayesian network method to whole-organism microarray data taken at ten time-points post exposure to a pyrethroid insecticide. The method corrects for both circadian patterns and mosquito ageing, which have previously been shown to be important in the insecticide resistance phenotype (Jones et al., 2012, Rund et al., 2016). The Bayesian network approach allows identification of key regulatory factors influencing the expression of transcripts in response to insecticide exposure. Based on validation experiments, we estimate that the inferred network has 70% precision, indicating strong concordance of experimental data to model prediction. The network is made freely available through a ShinyR application, allowing non-bioinformaticians to easily access and visualise the data. Several transcription factors are highlighted as potential key regulators in response to pyrethroid insecticide. This study demonstrates the importance and complexity of transcriptional control of insecticide response, which is likely to have cross species applicability due to relative conservation of transcriptional pathways (Hsia and McGinnis, 2003) and near total overlap of resistance mechanisms.

## Results

### Identification of transcription factors involved in insecticide resistance

Of the transcripts in the *Anopheles* microarray, approximately 4% are putative transcription factors, based on FlyMine.org AGAP homologs of Drosophila transcription factors found on FlyTF.org. As exploration of all possible transcription factor/transcript associations was not computationally feasible, the number of transcription factors had to be reduced to <50. Of the 559 total transcription factors, 44 were used in further analyses (Table 1). These transcription factors were selected based on resistance-associated GO term enrichments in transcription factor-transcript clusters (Zhang et al., 2018) found using a previously published library of microarray data comparing resistant and susceptible *Anopheles* species across Africa (Ingham et al., 2018). A number of these transcription factors have known roles in stress response in *Drosophila* (Table 1)*;* however, only *Maf-S, Met* and *Dm* have previously been linked with insecticide response in mosquitoes (Ingham et al., 2018, Ingham et al., 2017)*.* Of the transcription factors selected for analysis the following have been studied in mosquitoes: *p53* has been shown to respond to arboviral infection (Chen et al., 2017); *Rbsn-5* has been shown to be involved in egg shell formation (Amenya et al., 2010); *l(1)sc* is linked with sensory tissue development (Wülbeck and Simpson, 2002); *kaya*k is involved in salivary gland response to arboviral infection through *JNK* pathway activation (Chowdhury et al., 2020); *Hnf4* is linked to ecdysone and *Met* mediated lipid metabolism (Wang et al., 2017); *Cyc* controls the circadian ryhthm (Maliti et al., 2016); *REL1* and *REL2* are involved in immune response (Luna et al., 2006); *Kr-h1* is essential for egg development (Fu et al., 2020) and *Pan* is linked with chromatin changes upon *Plasmodium* infection (Ruiz et al., 2019).

**Table 1:** **List of Transcription Factors included in further analysis.** 44 Transcription factors included in the analysis with the dynamic Bayesian model, including VectorBase Transcript ID, Drosophila gene name, FBgn identifier, % identity (taken from VectorBase), putative function and network interactor summary KEGG/GO enrichment from this study (See S1 Table 1).

Transcript ID	Gene Name	Homolog	% Identity	Role in Drosophila	Citation	Network Enrichment
AGAP000057-RA	shaven (sv)	FBgn0005561	34.12	Sensory tissue development	Kavaler et al. 1999 (Kavaler et al., 1999)	None
AGAP000066-RA	Sox102F	FBgn0039938		Neuronal development, behaviour and Wnt signalling	Li et al. 2017 (Li et al., 2017)	mTOR and ECM-receptor interaction
AGAP000141-RA	CG31224	FBgn0051224	17.03	Unknown		Nuclear-related
AGAP000547-RA	Rbsn-5	FBgn0261064	42.29	Endosome assembly	Morrison et al 2008 (Morrison et al., 2008)	Polarity, Wingless
AGAP000646-RA	Diminuitive (Dm, dMyc)	FBgn0262656	13.21	Glucose and lipid metabolism, development	Parisi et al. 2013 (Parisi et al., 2013)	Sugar Metabolism, Miscellaneous Metabolism
AGAP000876-RA	achaete-scute complex (l(1)sc)	FBgn0002561	26.42	Neuronal development, dopaminergic neurons	Stagg et al 2011 (Stagg et al., 2011)	Cuticle-related, Neuroactive ligand-receptor
AGAP001093-RA	kayak (kay)	FBgn0001297	30.06	JNK signalling, wound healing, neuronal development	Ramet et al. 2002 (Rämet et al., 2002); Miotto et al. 2006 (Miotto et al., 2006)	RNA/DNA-related Processes
AGAP001156-RA	PSEA-binding protein 95kD (Pbp95)	FBgn0037540	13.89	Small nuclear RNA activating complex	Li et al 2004 (Li et al., 2004)	Cytochrome P450s, Signalling Pathways
AGAP001388-RA	Doublesex-Mab related 93B (dmrt93B)	FBgn0038851	41.61	Mouth development	Panara et al 2019 (Panara et al., 2019)	Taste/sense-related, Oxidoreductase Activity
AGAP001786-RA	Osa	FBgn0261885	36.83	EGFR signalling	Terriente-Feliz and de Celis 2009 (Terriente-Félix and de Celis, 2009)	GSTs
AGAP001994-RA	Brahma associated protein 111kD (Bap111)	FBgn0030093	40.1	Chromatin remodelling	Papoulas et al. 2001 (Papoulas et al., 2001)	Miscellaneous Metabolism, Cytochrome P450s, COEs
AGAP002082-RA	Squeeze (sqz)	FBgn0010768	35.47	Neuronal development	Terriente-Feliz et al 2007 (Félix et al., 2007)	Ligase Activity
AGAP002155-RA	Hepatocyte nuclear factor 4 (Hnf4)	FBgn0004914	52.85	Lipid mobilisation, glucose homeostasis and mitochondrial function	Palanker et al. 2009 (Palanker et al., 2009); Barry and Thummel 2016 (Barry and Thummel, 2016)	Glyoxylate Metabolism, Transcription Coactivator
AGAP002352-RB	p53	FBgn0039044	14.2	Genotoxic stress response	Brodsky et al. 2004 (Brodsky et al., 2004)	Carbon metabolism
AGAP002773-RA	Stripe (sr)	FBgn0003499		Muscle development	Lee et al. 1995 (Lee et al., 1995)	Steroid biosynthesis
AGAP002902-RA	Medea (Med)	FBgn0011655	52.42	Muscle development through BMP and dpp Pathways	Wisotzkey et al. 1998 (Wisotzkey et al., 1998)	Metabolism-related
AGAP002920-RA	CG17829	FBgn0025635	17.84	Unknown		Protein Complex Binding, DNA/RNA processes
AGAP002954-RA	Cell division cycle 5 (Cdc5)	FBgn0035136	63.63	Spliceosome	Herold et al. 2009 (Herold et al., 2009)	Notch Signalling, Apoptosis
AGAP003117-RA	Capicua (cic)	FBgn0262582	19.37	EGFR, Torso and TOLL signalling	Astigarraga et al. 2007 (Astigarraga et al., 2007); Papagianni et al.2018 (Papagianni et al., 2018)	Glycan degradation
AGAP003449-RA	Rootletin (Root)	FBgn0039152	46.08	Hearing, touch and taste	Chen et al. 2015 (Chen et al., 2015)	Steroid Biosynthesis, Receptor-related activity, Cytochrome P450s
AGAP003669-RA	Drop (Dr)	FBgn0000492	61.4	Eye and nerve development	Tearle et al. 1994 (Tearle et al., 1994)	Circadian Pathway
AGAP004864-RA	Protein on ecdysone puffs (Pep)	FBgn0004401	38.87	Hsp70 response through hnRNP complex	Hamann et al. 1998 (Hamann and Strätling, 1998)	Response to xenobiotics
AGAP004990-RA	Multiprotein bridging factor 1 (mbf1)	FBgn0262732	74.15	Co-activator to induce stress-response genes	Jindra et al. 2004 (Jindra et al., 2004)	Translation-related Processes
AGAP005437-RA	Inverted repeat binding protein 18 kDa (Irbp18)	FBgn0036126		Inhibitor of the conserved stress response protein dATF4/Crc	Blanco et al 2020 (Blanco et al., 2020)	Fatty Acid-related
AGAP005551-RA	Rabaptin-5-associated exchange factor for Rab5 (Rabex-5)	FBgn0262937	37.75	Ras pathway homeostasis	Yan et al. 2010 (Yan et al., 2010)	Apoptosis
AGAP005641-RA	CG9705	FBgn0036661	54.78	Sensory neurons	Iyer et al. 2013 (Iyer et al., 2013)	Protein Sorting, Response to DNA-damage
AGAP005655-RA	Cylce (Cyc)	FBgn0023094	35.25	Circadian rhythm	Rutila et al. 1998 (Rutila et al., 1998)	UGTs, Hormone Biosynthesis
AGAP006022-RA	Methoprene tolerant (Met)	FBgn0002723	21.2	Juvenile hormone binding	Jindra et al. 2015 (Jindra et al., 2015)	Oxidative Phosphorylation
AGAP006061-RA	Ken	FBgn0000286	5.92	JAK/STAT pathway	Arbouzova et al. 2006 (Arbouzova et al., 2006)	GTPase Activity, Vesicle-related, Actin-related
AGAP006392-RA	CG4617	FBgn0029936	38.58	Unknown		Autophagy
AGAP006601-RA	MEP-1	FBgn0035357	31.69	Chromatin remodelling	Reddy et al. 2010 (Reddy et al., 2010)	Peroxisome, CSPs
AGAP006642-RA	Defective proventriculus (dve)	FBgn0020307	47.98	Mitochondrial reactive oxygen species modulator	Baqri et al. 2014 (Baqri et al., 2014)	Behavioural-related, Neuron-related
AGAP006736-RA	Sugarbabe (sug)	FBgn0033782	28.24	Regulation of lipid and carbohydrate metabolism	Varghese et al. 2010 (Varghese et al., 2010)	P450, IMD-pathway
AGAP006747-RA	Relish (REL2)	FBgn0014018	24.12	Immune response	Dushay et al. 1996 (Dushay et al., 1996)	Transferase, Dendrite-related, CSPs
AGAP009444-RA	Suppressor of variegation 205 (Su(var)205)	FBgn0003607	23.47	Hsp70 response through activation of euchromatic genes	Piacentini et al. 2003 (Piacentini et al., 2003)	Ribosome-related, Hippo signalling
AGAP009494-RA	Ets at 21C (Ets21C)	FBgn0005660	34.25	Stress inducible transcription factor through JNK	Mundorf et al. 2019 (Mundorf et al., 2019)	Behaviour-related, Neuronal, JAK/STAT
AGAP009515-RA	REL1	FBgn0260632	38.96	Toll pathway	Gross et al. 1999 (Gross et al., 1999)	Vesicle-related Transport, Mitophagy, Toll pathway
AGAP009662-RA	Kruppel Homolog 1 (Kr-h1)	FBgn0028420	36.47	20-hydroxyecdysone linked	Pecasse et al. 2000 (Pecasse et al., 2000)	TCA-cycle
AGAP009676-RA	Chameau (chm)	FBgn0028387	34.66	JNK signalling	Miotto et al. 2006 (Miotto et al., 2006)	Transmembrane Signalling, Behavioural-related, Neuronal
AGAP009888-RA	CG33695	FBgn0052831	53.3	Unknown		Hippo Signalling, COEs
AGAP009899-RA	klumpfuss (klu)	FBgn0013469	42.86	Cell death, mitochondrial function, EGFR signalling	Protzer etl al. 2008 (Protzer et al., 2008); Chen et al. 2008 (Chen et al., 2008)	Morphogenesis, Drug Metabolism, UGTs, GSTs
AGAP009983-RA	Net	FBgn0002931	35.88	EGFR signalling	Terriente-Feliz and de Celis 2009 (Terriente-Félix and de Celis, 2009)	MAPK/Notch Signalling
AGAP010405-RA	Maf-S	FBgn0034534	63.7	Reactive oxygen species stress response	Misra et al. 2011 (Misra et al., 2011)	Respiration-related, Insulin-related
AGAP012389-RA	Pangolin (Pan)	FBgn0085432	24.47	Wingless signalling	Brunner et al. 1997 (Brunner et al., 1997)	Wnt-signalling, COEs

### Modelling the insecticide response network

To explore the role of the identified transcription factors in insecticide response, a previously generated time course experiment comparing pyrethroid exposed and unexposed *Anopheles coluzzii* was used (Ingham et al., 2020). This dataset was then used to model the gene regulatory relationships using a dynamic Bayesian network (DBN) approach (Dondelinger et al., 2013) which infers the regulators of each transcript from the set of selected transcription factors using the time-course of log-fold changes compared to the unexposed baseline measurement, correcting for ageing and circadian rhythms. A Markov chain Monte Carlo (MCMC) algorithm was used to draw samples from the posterior distribution of the network model given the data, and associations were then ranked between target genes and transcription factors using the marginal posterior probability of the corresponding edge (defined as a predicted transcription factor – transcript association) in the network. Since experimental validation of all discovered edges is prohibitively expensive, an important consideration was how many associations needed to be tested in order to establish the validity of the network inference approach. A simulation study was performed under the assumption that the number of genes regulated by each transcription factor follows a Poisson distribution with parameter λ=10. We showed that under some assumptions (see Materials and Methods) testing 4 regulatory relationships for each of 7 transcription factors has a 70% chance of obtaining an estimate of the precision that falls within 10% of the true precision, and a 95% chance of obtaining an estimate that falls within 20% of the true precision. For 5 transcription factors with 4 regulatory relationships, this still gives a 65% chance of an estimate within 10%, and a 90% chance of an estimate within 20% of the true precision.

The model was validated using quantitative PCR to confirm the interactions predicted by the model. Successful dsRNA mediated knock down was performed on 5 transcription factors, these showed knock down 48-hours post insecticide exposure (Supplementary Figure 1); the single time point used for model confirmation (Supplementary Figure 1, Supplementary Table 1). Four transcript interactors were chosen randomly for each transcription factor based on a posterior probability of > 0.1. To determine the change in transcript expression post-exposure and to determine whether predicted interactors were influenced by the knock down of the stated transcription factor 2 comparisons were made: (i) GFP-injected exposed vs GFP-injected unexposed and (ii) Exposed transcription factor knockdown compared to exposed GFP-injected for the two comparisons respectively (Supplementary Table 1). Of the 16 interactors (4 transcription factors x 4 interactors), 11 demonstrated concordance with the model, showing a substantial change in expression due to transcription factor knockdown, indicating 69% model precision (Figure 2, Supplementary Table 1).

### Network Overview

In order to determine what the optimal cut-off for the marginal posterior probability values should be, a permutation test was performed whereby the observed log-fold values for one of the 44 transcription factors are randomly permuted, so that the original time associations were no longer present (Appendix 1). Any association between this transcription factor and the target gene would then be purely due to chance. This process was then repeated 500 times, inferring the edges for all 44 transcription factors each time. The resulting marginal posterior probability values were then analysed for the randomised transcription factor and showed that a threshold of 0.39 was sufficient to only produce one false positive out of 500 randomizations, or a false positive rate of 0.002 (Appendix 1), which resulted in assignment of 5136 transcripts to the 44 transcription factors.

**Figure 1:**
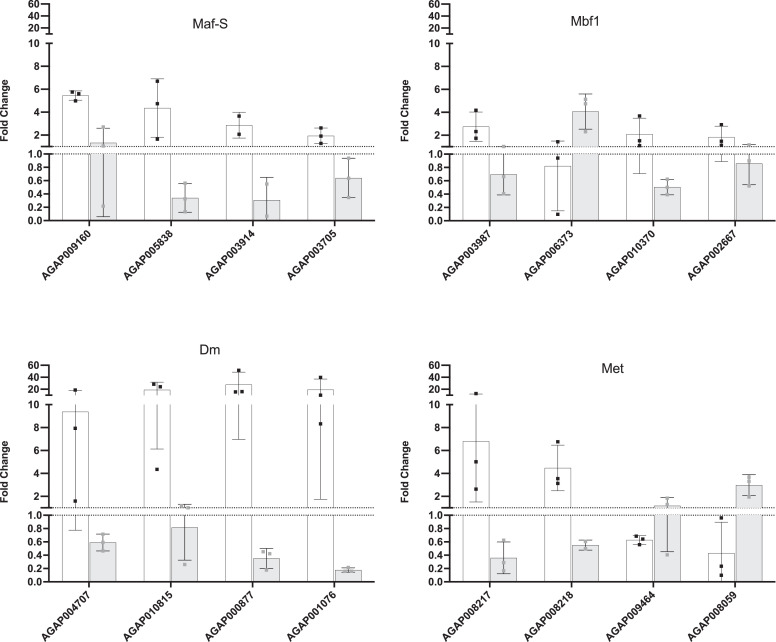
**Model validation.** mRNA fold change (y-axis) of each transcript (x-axis) for each transcription factor showing knockdown 48-hours post-deltamethrin exposure. White bars show qPCR results from GFP-injected exposed mosquitoes (48-hours post exposure) compared to GFP-injected unexposed mosquitoes (48-hours post injection) to show induction effect in absence of treatment and grey bars show transcription factor-injected exposed (48 hours post exposure) vs GFP-injected exposed mosquitoes (48-hours) to demonstrate the effect of transcription factor knockdown. Error bars show standard deviation.

The complete network using a posterior probability cut-off of 0.39 is displayed in Figure 1. Due to the constraints imposed by this model on number of parent nodes tested, simple network descriptive data was generated only for edges from the selected transcription factors. The average edge count was 118.48±179.62 demonstrating high variance in connectivity as seen in Figure 1 with a range of 8 associations to 951. 23 transcription factors are network hubs, defined as nodes with a high number of associations (>50) (Table 2), including *Dm, Met* and *Maf-S* all previously linked with the insecticide resistance phenotype (Ingham et al., 2018, Ingham et al., 2017) and *mbf1* a stress response transcription factor (Jindra et al., 2004). To enable the network to be freely accessible an application NetworkVis has been written in ShinyR (Chang et al., 2017) and is available online (https://github.com/VictoriaIngham/NetworkVis_TimeCourse; Supplementary Table 2) with all associated data. Users can manually select a posterior probability cut-off between 0.1-0.8, select and rearrange nodes and edges in the network and identify *a priori* transcription factors through visual means rather than working with a large text file.

**Figure 2:**
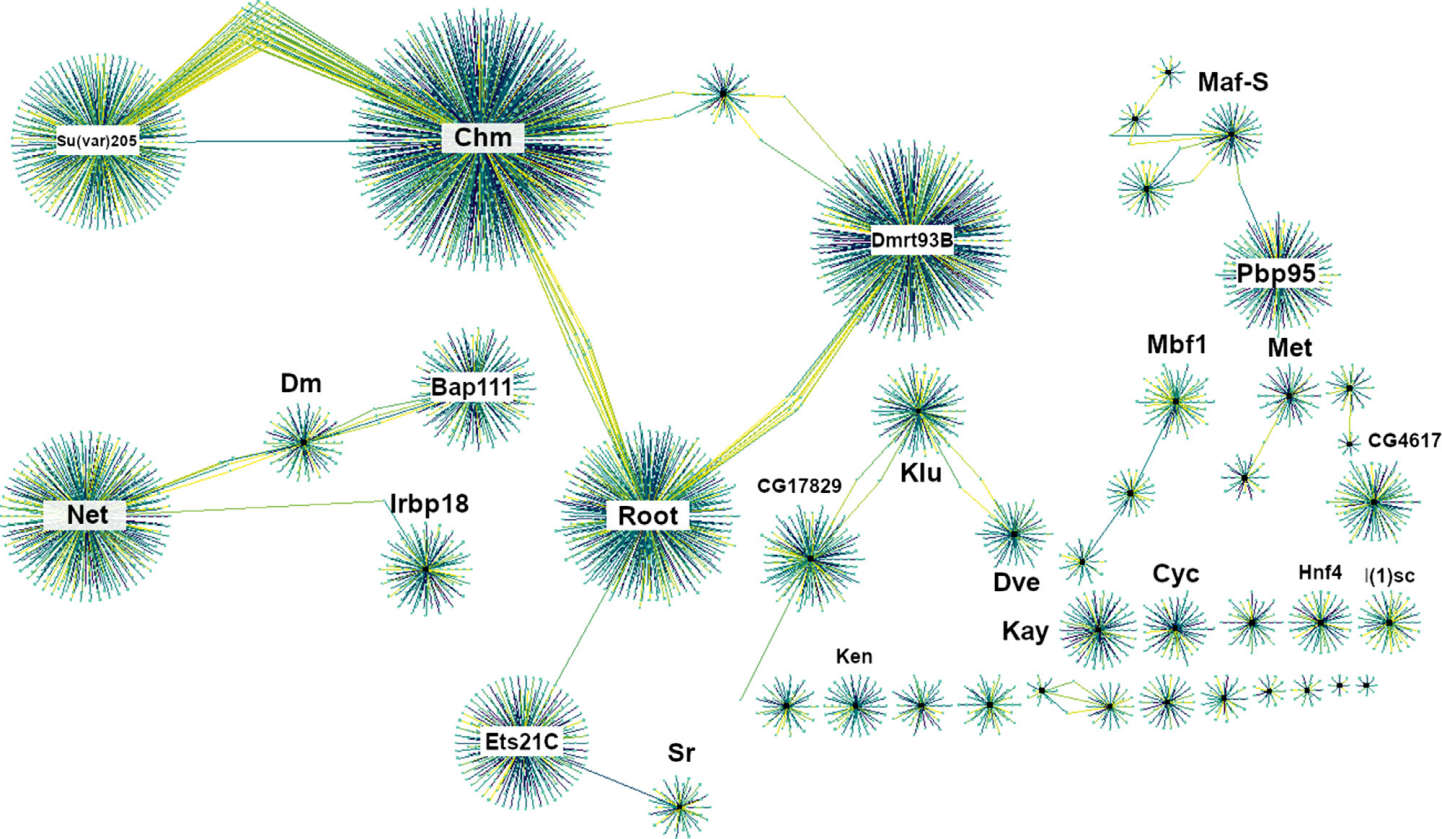
**Network overview.** Emboldened black circles represent all 44 transcription factors, with grey nodes representing associated transcripts. Directed edges are coloured on posterior probability gradient from yellow (0.39) through green (0.5) to dark blue (0.97). High posterior probability indicates higher confidence in the interaction. The 23 hub transcription factors, with > 50 associations are labelled.

**Table 2:** **Transcription factor hubs.** Identifier, gene name and number of associations for 23 transcription factor hubs within the network.

Transcription Factor	Name	Number of associations
**AGAP009676-RA**	Chm	951
**AGAP001388-RA**	dmrt93B	535
**AGAP009444-RA**	Su(var)205	447
**AGAP003449-RA**	Root	447
**AGAP009983-RA**	Net	399
**AGAP009494-RA**	Ets21C	227
**AGAP001994-RA**	Bap111	201
**AGAP001156-RA**	Pbp95	185
**AGAP002920-RA**	CG17829	145
**AGAP009899-RA**	Klu	118
**AGAP005437-RA**	Irbp18	113
**AGAP006392-RA**	CG4617	98
**AGAP000646-RA**	Dm	91
**AGAP001093-RA**	Kay	87
**AGAP004990-RA**	Mbf1	72
**AGAP006642-RA**	Dve	69
**AGAP005655-RA**	Cyc	66
**AGAP010405-RA**	Maf-S	64
**AGAP002155-RA**	Hnf4	60
**AGAP006601-RA**	Ken	58
**AGAP000876-RA**	l(1)sc	57
**AGAP006022-RA**	Met	53
**AGAP002773-RA**	Sr	51

Enrichment analysis was run for every transcription factor and associated interactors for all GO term categories (Ashburner et al., 2000), KEGG pathways (Kanehisa and Goto, 2000), gene families previously associated with resistance (Balabanidou et al., 2016, Müller et al., 2008, Voice et al., 2015, Ingham et al., 2018, Ingham et al., 2019) and Reactome pathways based on *Drosophila* homology (Jassal et al., 2020) (Table 1, Supplementary Table 3).

GO enrichments were present for 21/44 of the transcription factors across all ontology categories (Molecular Function, Cellular Component and Biological Process). A large number of GO terms were significant across different transcription factor interactions analysed; however, the terms were largely non-overlapping indicating that the transcription factors are playing differing roles in insecticide response (Supplementary Table 3, Supplementary Figure 2). Seven GO terms (dendrite, dendritic tree, somatodendritic compartment, transmembrane signalling receptor activity, signalling receptor activity, response to drugs) were significant across four transcription factors and relate to terms clearly involved in stress response and associated behavioural changes.

KEGG enrichments were present for 39/44 transcription factors (Supplementary Table 3, Supplementary Figure 3), again there was minimal overlap in the enriched pathways, in agreement with the divergent enriched GO terms. One KEGG pathway was significant for six transcription factor associations (neuroactive ligand-receptor interaction) and two terms were significant for four transcription factor associations (insect hormone biosynthesis, other glycan degradation).

Given our *a priori* knowledge of insecticide resistance, enrichment analysis was also carried out for detoxification gene families, the cuticular hydrocarbon synthesis pathway and chemosensory proteins; three well described resistance mechanisms (Balabanidou et al., 2016, Müller et al., 2008, Voice et al., 2015, Ingham et al., 2019). Enrichments for these families occurred in 20/44 transcription factors with cytochrome p450s being significantly enriched in eight, GSTs in four, UGTs in three, COEs in eight, chemosensory proteins in two and the cuticular hydrocarbon pathway in three (Supplementary Table 3, Supplementary Figure 4). Reactome enrichment was also carried out, with significance for at least one pathway in 21/44 of the transcription factors (Supplementary Table 3, Supplementary Figure 5).

Taken together, these data indicate that the applied DBN is successfully capturing differing roles of the transcription factors in insecticide exposure response and the enrichment of a large number of *a priori* detoxification candidates indicates we are successfully capturing transcription factors controlling metabolic response to insecticide exposure.

### Key transcriptional regulators of insecticide response

Transcription factors that have previously been implicated in insecticide resistance or stress response and those that have interactors which show a clear functional enrichment from the above analysis are described in greater detail below.

#### Chameau

*Chameau* (*Chm*, AGAP009676-RA) is the transcription factor with the highest number of interactors at 951. *Chm* interactors are strongly enriched in transmembrane signalling activity (p = 1.32e-8), sensory perception (p = 3.39e-6) and chemosensory behaviour (p = 8.74e-4). 26 other transcription factors interact directly with *Chm* including Abd-B (AGAP004664-RA) including a known Drosophila interaction and *so* (AGAP011695-RA), *Fer3* (AGAP003756-RA), *disco* (AGAP01106-RA), *C15* (AGAP003674-RA), *zfh1* (AGAP000779-RA), *hkb* (AGAP004517), all known secondary interactors. 14 interactors have posterior probabilities of >0.90, including *fringe* (AGAP006439-RA) a gene involved in regulating the Notch signalling pathway (Moloney et al., 2000), which is significantly enriched in *Chm* interactors (p = 0.012) and *Roquin* (AGAP007114-RA) a protein that translocates to stress granules on chemical induced toxicity (Athanasopoulos et al., 2010, Voßfeldt et al., 2012).

#### Diminuitive

*Diminuitive* (*Dm*, AGAP00646-RA) is a central network hub with 91 interactors and its interactors are enriched in multiple KEGG pathways such as N-glycan biosynthesis, protein processing in the endoplasmic recticulum and starch and sucrose metabolism (Supplementary Table 3) (Martinez-Torres et al., 1998, Ingham et al., 2017, Nagy et al., 2013, Kappes et al., 2011). Previous work has demonstrated that attenuating *Dm* expression in *An. gambiae* results in significantly higher mortality post-pyrethroid exposure (Ingham et al., 2018); this role is underlined by significant enrichment of detoxification gene families in this cluster including the COEs (p = 0.031) and ABCs (p = 7.2e-3) (Wilding et al., 2014, Riveron et al., 2014). Interestingly, the ABCs in this network belong to the ABCB family of transporters, which are known as multi-drug transporters and are implicated in insecticide resistance in *Drosophila* and *Anopheles* (Gellatly et al., 2015, Pignatelli et al., 2018)*. Dm* also interacts with *Bap111*, whose network is enriched for fatty acid degradation and cuticular hydrocarbon synthesis and contains the cytochrome p450 *CYP4G17*, previously linked with cuticular thickening in resistant mosquitoes (Balabanidou et al., 2016). (Balabanidou et al., 2016)

#### Doublesex-Mab related 93B

*Doublesex-Mab related 93B (dmrt93B*, AGAP001388-RA) is the second most well-connected node with 535 interactors. *Dmrt93B* is enriched in multiple GO-terms related to xenobiotic metabolism, including oxidoreductase activity (p = 7.7e-3), heme-binding (p = 2.6e-4) and monooxygenase activity (p = 0.014) as well as being highly enriched in the *a priori* detoxification gene families; cytochrome p450s (p = 5.53e-6), COEs (p = 0.023) and GSTs (p = 0.029). Taken together, these enrichments indicate that *dmrt93B* is playing a central role in the response of metabolic transcripts to insecticide exposure. Although not showing enrichment in a related term, 14 cuticular proteins are present in this interactome, one of which *CPLCP11* (AGAP009758-RA) has been shown to be up-regulated in resistant mosquitoes compared to susceptible (Balabanidou et al., 2019) and another, *CPR133* (AGAP009872-RA), has the highest posterior probability (0.93).

#### *Met* and *Maf-S*

Both *Maf-S* (AGAP010405-RA) and *Met* (AGAP006022-RA) have previously been shown to have important roles in insecticide response (Ingham et al., 2018, Ingham et al., 2017). In the absence of insecticide exposure, attenuation of expression of these transcripts demonstrated that both influenced the expression of key pyrethroid metabolisers such as *CYP6M2, CYP6Z2, CYP6Z3, CYP6P4, GSTD1* and *CYP9K1* (Yunta et al., 2019)(Ingham et al., 2017). *Met* interacts with *CYP6Z2* which is amongst the most strongly induced p450s in the dataset with a marginal posterior probability of 0.88. Interestingly, *Maf-S* shows enrichment in ABC transporters and terms related to ATP production, indicating *Maf-S* may play a role in changes in metabolism, which is a striking feature of this dataset. *Met* shows enrichment in glycolysis, potentially indicated an overlap in the function of these transcription factors, which would be in agreement with the *Maf-S* knockdown microarray which identified *Met* as a direct interactor. (Murata et al., 2015, Cornelissen et al., 2018)

#### Mbf1

*Multiprotein bridging factor 1* (*mbf1*, AGAP004990) has 119 interactors and is enriched for GO terms related to the ribosome (p = 0.026) and RNA binding (p = 0.048) and is highly enriched in the KEGG ribosome (p = 4e-4). The role of *mbf1* in *Drosophila* involved translocation to the nucleus upon cellular stress, where it serves as a co-activator of stress response genes (Jindra et al., 2004); despite this role no enrichment for detoxification transcripts is seen in the predicted *mbf1* associations. However, 1 chaperone protein (*CCT6*) and an oxidative stress sensing protein (AGAP000705-RA) are present in this network. AGAP002667 has the highest posterior probability in the network (0.84) and encodes the homolog of *Drosophila Tctp* which is necessary for genomic stability under genotoxic stress (Hong and Choi, 2013).

(Taylor-Wells et al., 2015, Zhong and Wu, 2004, Ng and Luo, 2004, Koch et al., 2008, Schaefer et al., 2001, Varghese et al., 2010, Musselman et al., 2018, Bharucha et al., 2008, Beller et al., 2010)

## Discussion

In this study, we apply a dynamic Bayesian network approach to whole transcriptome time-course data post-sublethal exposure of *An. coluzzii* to the pyrethroid insecticide deltamethrin (Ingham et al., 2020). The modified DBN model employed here allows correction for not only circadian rhythms, but also for mosquito ageing, a critical variable in the resistance status (Jones et al., 2012). Interactions predicted by this model were then validated *in vivo*, demonstrating high model confidence, with 70% precision. The high model precision and the overlapping biological functions with known transcription factors in *Drosophila* demonstrates the utility of this approach in assigning transcription factor function. Furthermore, this study highlights the potential for use of this methodology across multiple species of interest in which lower resolution time points are more feasible than those seen in model organism studies. Potential applications of this methodology could include exploring transcriptional regulation of pesticide response in other pest species or exposing the same species to additional stressors to distinguish between transcription factors involved in general and insecticide induced stress response.

In this study we highlight 44 transcription factors with putative roles in response to sublethal pesticide exposure, 41 of which have not previously been linked to insecticide resistance. Of the 6585 transcripts differential in the data set used, 5136 transcripts were assigned associations with these 44 transcription factors, using a posterior probability cut-off of >0.39. The assignment of 78% of the overall responsive transcripts is likely due to necessity of reducing the number of transcription factors to less than 50 transcripts and responsive transcripts being regulated by other mechanisms such as non-coding regulatory machinery. The transcription factors selected here for further analysis were identified by applying an SILGGM model (Zhang et al., 2018) to 28 insecticide resistant vs susceptible microarray datasets performed on the *Anopheles gambiae* species complex collated by Ingham et al. (Ingham et al., 2018) and exploring enrichments of co-correlated transcripts; this represents a confounding aspect of this methodology as these transcripts are constitutively overexpressed and not induced by insecticide exposure due to the nature of the transcriptomic designs.

Of the 44 transcription factors, 3 had previously been linked with insecticide resistance in *Anopheles* mosquitoes and just 11 had been previously studied in mosquito species in any context (Ingham et al., 2018, Ingham et al., 2017, Ruiz et al., 2019, Chen et al., 2017, Amenya et al., 2010, Wülbeck and Simpson, 2002, Chowdhury et al., 2020, Wang et al., 2017, Maliti et al., 2016, Luna et al., 2006, Fu et al., 2020). All but 4 of these transcription factors have a well-defined role in *Drosophila*. Using a posterior probability cut off of >0.39, the number of associations showed high levels of variation with an average edge count of 118.48±179.62, potentially demonstrating differential importance in insecticide response, with those transcription factors with a high number of edges or high network connectivity being more important. 23 transcription factors were designated as transcript ‘hubs’ based on high levels of network interconnectivity (>50 edges).

Enrichment analysis was performed for all transcription factors in the network, using GO Terms, KEGG Pathway, Reactome and *a priori* transcript families with links to resistance. Interestingly, the overlap of enriched terms was low, indicating that each transcription factor may play a differing role in the response to insecticides. 20 transcription factors show enrichments in *a priori* gene families; this may be an unsurprising feature of this dataset given the obvious change in expression across multiple members of these families within this dataset and their documented importance in insecticide metabolism (Ingham et al., 2020). GO terms enriched across multiple transcription factors include terms expected in an insecticide response, response to drugs, drug metabolism, sensory perception of chemical stimuli and ABC transporters. The former two enrichment terms are in agreement with the well-established dogma that up-regulation of members of the cytochrome p450 class play a direct role in increasing the rate of insecticide metabolism (Ingham et al., 2018, Yunta et al., 2019). (Oliver and Brooke, 2016, Wang et al., 2016). Interestingly, changes to the respiratory pathway through alterations to the oxidative phosphorylation pathway also appears across multiple transcription factors and is a striking feature of this dataset (Ingham et al., 2020).

To cross-validate the function of these transcription factors, their known functions in the model organism *Drosophila* were explored. Despite the differences in hypotheses explored in this study and the available data in discerning *Drosophila* pathways, there were clear overlaps in transcription factor roles and associations. For example, *Dm* is known to play a role in lipid and glucose homeostasis in *Drosophila* (Parisi et al., 2013) and here, the associations are enriched in the KEGG pathways starch and sucrose metabolism; this is similar to *dmrt93B* which is involved (Palanker et al., 2009) mouth part development and is enriched in the GO term related to taste receptor activity (Panara et al., 2019). Several further transcription factors show overlap with *Drosophila* function, including *Pep* which is involved in stress response through activation of Hsp70 (Hamann and Strätling, 1998, Varghese et al., 2010), *dve* which is involved in reactive oxygen species modulation (Baqri et al., 2014), *Ets21C* which is a stress-inducible transcription factor (Mundorf et al., 2019), *klumpfuss* whose role is related to morphogenesis in the central nervous system (Melcher and Pankratz, 2005), REL1 which is implicated in the TOLL pathway (Gross et al., 1999, Murata et al., 2015) and *Chm* is a known modulator of the stress responsive JNK pathway with a role in sensory cell fate (Melcher and Pankratz, 2005). (Wang et al., 2017, Ruiz et al., 2019)

This study provides not only previously unreported transcription factors that are involved in the transcriptional response to pesticide exposure but demonstrates the utility of applying a model-based approach to lower-resolution time course data in ascertaining these associations. Here, six transcription factors and their interactomes were delineated as hub transcripts within the network, all of which have either been previously linked to resistance or stress response in *Anopheles* (*Dm, Maf-S* and *Met*) (Ingham et al., 2018, Ingham et al., 2017) or *Drosophila (mbf1*) (Jindra et al., 2004) or are highly significantly enriched for clear functions (*chameau* and *dmrt93B)*. These transcription factors are likely to be involved in different facets of insecticide response and represent pathways that should be further explored. The modelling approach taken here, which accounts for both circadian patterns and ageing, two key determinants in pesticide resistance, can be applied widely to other pest or vector species. Using this approach will provide invaluable information on changes to pest biology post-pesticide exposure and will elucidate new pathways to characterise and target to tackle the ongoing threat of pesticide resistance.

## Materials and Methods

### Microarray Experiments

Microarrays were taken from (Ingham et al., 2020) and consist of deltamethrin exposed mosquitoes compared to unexposed at the following time points post-exposure: 0 minutes, 30 minutes, 1 hour, 2 hours, 4 hours, 8 hours, 12 hours, 24 hours, 48 hours and 72 hours. To account for ageing effects, a twin time course was performed using age matched females that were unexposed to insecticide at the following time points: 8 hours, 12 hours, 24 hours, 48 hours and 72 hours. All mosquitoes within one experimental time course came from the same generation. Experimental data is available on exposure time course (E-MTAB-9422) and ageing time course (E-MTAB-9423). Analysis was performed as previously described.

### Transcription factor identification

To identify relevant nodes for the Bayesian analysis, 28 microarray datasets encompassing resistant vs susceptible members of the *Anopheles gambiae* species complex were used from Ingham et al. 2018 (Ingham et al., 2018). A de-sparsified node-wise scaled lasso (Janková and van de Geer, 2017, Janková and van de Geer, 2018) implemented in the R package SILGGM (Zhang et al., 2018), was used to infer the gene network. This method employs L1-regularisation to preserve sparsity in the estimated network. For the L1-regularisation, the default value of the tuning parameter λ, was used: √log(p)/n, where p is the number of variables and n is the number of samples. The resultant Gaussian graphical model produced a 14079 × 14079 file for every possible interaction in the transcriptome. Each interaction had an associated p-value for precision (Supplementary Table 4). A cut-off value of p ≤ 0.1 was used to filter all interactions to prevent loss of potentially interesting transcription factors due to the differing experimental design of the data set used. Annotated Drosophila transcription factors were downloaded from FlyTF (Adryan and Teichmann, 2006) (http://flytf.gen.cam.ac.uk/) and *Anopheles* homologs identified using FlyMine (Lyne et al., 2007) (https://www.flymine.org) using the analyse input box, and then selecting *An. gambiae* homologs, resulting in 559 putative transcription factors; all 559 were then extracted from the inferred network with all associated putative co-correlating transcripts. clusterProfiler (Yu et al., 2012) and AnnotationForge (Carlson and Pagès, 2019) were used to perform GO enrichments using an Anopheles database built from PEST/VectorBase (Giraldo-Calderón et al., 2014) on Biological processes on transcription factors with > 10 interactors. Transcription factors enriched in the following character patterns were extracted: ‘stress’; ‘oxi’; ‘lipid’; ‘behaviour’; ‘response’; ‘fat’; ‘sensory’ and ‘ATP’ leading to 54 transcription factors. The terms were chosen based on previous knowledge of the resistant mechanisms present in *An. coluzzii* mosquitoes as detailed in the introduction (Balabanidou et al., 2016, Müller et al., 2008, Voice et al., 2015, Ingham et al., 2018, Ingham et al., 2019, Ingham et al., 2020) and ‘ATP’ due to a striking change in metabolism observed in this data set (Ingham et al., 2020). The transcription factors were further filtered on at least 50% of the transcripts in the cluster generated by SILGGM being differentially expressed in at least 1 time point within the time course datasets with an adjusted p value of ≤ 0.05. This procedure resulted in 44 transcription factors being retained. To estimate the impact of transcription factor choice on the network inference, target genes with a marginal posterior probability of >0.75 of having an association with at least one of the transcription factors in the dynamic Bayesian network analysis were selected and the model was re-run using a random selection for 25% of the transcription factors (11/44). The difference in marginal probability of the associations was then analysed. As the majority of differences are <0.2, the 0.39 cut-off used here would still correctly identify the associations with > 0.75 marginal posterior probability in the original analysis (Supplementary Figure 6).

### Network reconstruction using Dynamic Bayesian Networks

Dynamic Bayesian networks (DBNs) (Dondelinger et al., 2013) were used to identify directed associations between the transcription factors and putative regulated genes. A dynamic Bayesian network defines a graphical model for the dynamics of time series data, where the gene expression $X_{i}\left(t\right)$ of gene i at time t depends on the gene expression $X_{j}\left(t\right)$ of all transcription factor genes j at time t−δ. The relationship can be described by the following auto-regressive linear regression:

$$X_{i}\left(t\right)=a_{0}+\sum_{j\in TF}a_{j}X_{j}\left(t-\delta \right)+\varepsilon$$

where $\varepsilon N(0,\sigma_{i}^{2}),\wedge T$ *Fistheseto f indices representing the transcription factors*. To impose regularisation, we assumed truncated Poisson priors on the number of regression parameters $a_{j}$ that are non-zero:

$$P\left(s_{i}\vee \lambda \right)\propto \frac{\lambda^{s_{i}}}{s_{i}!}I\left(s_{i}<s_{max}\right)$$

where $s_{max}$ is the maximum number of transcription factors regulating a single gene. We set $s_{max}=5$. Conditional on $s_{i}$, the number of non-zero transcription factor-gene associations, the prior on the set of transcription factors for a given gene is simply a uniform distribution.

Inference of the network structure can be done via a Markov Chain Monte Carlo algorithm, with discrete moves allowing for adding and removing edges during the sampling. Convergence was assessed by running each MCMC chain twice from independent starting points and comparing the marginal posterior edge probability estimates. We ran the MCMC algorithm for 500,000 iterations, discarding the first quarter as burn-in, which ensured good convergence across all target genes. For full details on the model and inference procedure, please see Appendix 1. Note that here we employ a simplified version of the model in (Dondelinger et al., 2013) which does not use a changepoint model or information sharing priors.

Prior to applying the network inference model, we pre-processed the log-fold change data by first averaging the values for genes with multiple probes to obtain one measurement per gene. We then employ LOESS estimation (Cleveland et al., 1992), a local regression method which fits low-degree polynomials to subsets of the data, to interpolate the time points at t−δ, where we choose δ=0.5 hours as the time interval. Interpolation is necessary, as the DBN method requires equal time intervals between each pair of measurements to estimate consistent associations.

We further extend the model to correct for circadian rhythms and ageing effects in the gene expression levels. For the circadian rhythm correction, we assume that all circadian rhythms have a period of 24 hours, and augment the design matrix **X** = $\left\{X_{1}\left(t\right),...,X_{p}\left(t\right)\right\}$ with two additional columns for the sine and cosine functions of a 24-hour periodic signal:

$$X_{sin}\left(t\right)=sin\left(2\pi t/24\right),X_{cos}\left(t\right)=cos\left(2\pi t/24\right)$$

The resulting harmonic regression model with automatically correct for circadian rhythms, including under phase shift, by adding the periodic signal as a parent in the network, while non-periodic genes will remain unconnected to this signal.

Similarly, we add additional columns for the data arising from the ageing controls to correct for the effect of ageing. Note that here we only have data starting from 8 hours, so earlier time points will be uncorrected, and the corresponding values in the design matrix will be set to zero. The final autoregressive model looks as follows:

$$X_{i}\left(t\right)\;=\;a_{0}+\sum_{j\in TF}\left\{a_{j}X_{j}\left(t-\delta \right)\right\}\;+\;b_{sin}X_{sin}\left(t\right)\;+\;b_{cos}X_{cos}\left(t\right)\;+\;cX_{i,ageing}\left(t\right)+\varepsilon$$

where $X_{i,ageing}\left(t\right)$ is the log-fold change of the ageing controls.

We summarize the results of the DBN analysis using the marginal posterior probability of each transcription factor – target gene association, which can be calculated by obtaining samples from the converged Markov chain and averaging over the presence/absence status of each edge. In order to determine a sensible threshold for the marginal posterior probability that keeps the false discovery rate low, we implement the following permutation test to estimate the posterior probabilities under the null hypothesis of no associations: for each of n=500 iterations, we randomly permute the log-fold changes for one transcription factor. Any associations with the target gene should then be entirely by chance. Taking all n=500 samples of the null distribution obtained in this way, we determine that a threshold of 0.39 was sufficient to only produce one false positive out of 500 randomizations, or a false positive rate of 0.002. Further detail of the model can be found in Appendix 1. The network was displayed using Cytoscape (Shannon et al., 2003).

To estimate how the computational time needed scales with the number of transcription factors, we repeatedly selected 10 target genes and p transcription factors, where p∈(5,100). Network inference using EDISON was then performed on a computational cluster with two Intel Xeon E5-2660 v4s, which have 14 physical cores running @ 2.00GHz each, and 256 GB of RAM, and the resulting computational time is recorded. All MCMC chains for the network inference algorithm are run for 500,000 iterations (Supplementary Figure 7).

### NetworkVis App

The NetworkVis app and associated data can be downloaded on Github (https://github.com/VictoriaIngham/NetworkVis_TimeCourse) and installed as described. ShinyR (Chang et al., 2017) was used to create a user interface, both VisNetwork (Almende et al., 2018) and igraph (Csardi and Nepusz, 2006) were used to allow dynamic selection of nodes and edges, and to display the network.

### Enrichment analysis

Enrichment analysis was performed using clusterProfiler (Yu et al., 2012) and a custom *Anopheles* database produced using AnnotationForge (Carlson and Pagès, 2019). GO term and KEGG enrichments were performed using a Benjamini-Hochberg corrected p value cut-off of ≤ 0.05 with transcription factors > 10 interactions. Clusters of each transcription factor were compared using the compareCluster function using default parameters, Benjamini-Hochberg correction and a full background geneset from org.Agambiae.eg.db built from the PEST assembly; these were then displayed using Cytoscape (Shannon et al., 2003). Enrichment analysis on individual gene families were performed using a hypergeometric test with the phyper function in R; significance was considered when p ≤ 0.05. Reactome analysis was also performed using a hypergeometric test with p ≤ 0.05; *Drosophila* pathway membership was downloaded from Reactome.org (https://reactome.org/) (Jassal et al., 2020) for each pathway of interest, FlyMine (Lyne et al., 2007) was then used to convert these to *Anopheles* homologs. FlyBase (Consortium, 2003) was used to determine functions of homologs throughout the analysis. We applied the Benjamini-Hochberg correction for multiple testing outputs of the hypergeometric test.

### Validation of Network

We first performed a simulation study to determine the number of associations that need to be tested experimentally in order to obtain an accurate estimate of the precision of our network inference method. We made the following assumptions: (i) The mean number of gene regulated by each transcription factor is 10, and the actual number of regulated genes follows a Poisson distribution; (ii) The rate of true positives (correctly predicted associations) of our network is 0.75, and the rate of true negatives (correctly predicted non-associations) is 0.997; this results in a precision of ∼0.56 and a recall of ∼0.72 (Appendix 2); (iii) Transcription factors and regulated genes to test are selected randomly and (iv) The qPCR knockdown test is 100% accurate. The results of the simulation study can be found in Appendix 2. We concluded that testing 4 regulatory relationships for 7 transcription factors has a 70% chance of obtaining an estimate of the precision that falls within 10% of the true precision, and a 95% chance of obtaining an estimate that falls within 20% of the true precision.

In order to choose associations for validation, we then chose interactors by extracting the transcription factor of interest and associated transcripts from the results of the network inference. Transcripts were listed as 1 to n based on posterior probability in descending order. A random number generator was then used to select 4 transcripts for validation from 6 transcription factors chosen based on previous knockdown in the case of *Maf-S, Met, Dm* or through a random number generator.

### Mosquito Rearing

The *An. coluzzii* VK7 colony reared and profiled at Liverpool School of Tropical Medicine were used for all experiments (Williams et al., 2019). VK7 are a highly pyrethroid resistant population originating from Vallée de Kou, Burkina Faso (Toé et al., 2015). They have been reared at LSTM since 2014 under pyrethroid selection pressure (Williams et al., 2019). All mosquitoes used were reared under standard insectary conditions of 27°C and 70-80% relative humidity under a 12:12 photoperiod and are presumed mated.

### dsRNA knockdown

RNAi was performed using 7 transcription factors based on previous publication of knockdown (Maf-s, Met, Dm (Ingham et al., 2018, Ingham et al., 2017)) or through random selection using a random number generator (Med, Pan, l(1)sc, mbf1) (Supplementary Table 5). PCR was performed on 3-day old VK7 unexposed cDNA using Phusion® High-Fidelity DNA Polymerase (Thermo Scientific) following manufacturer's instructions and primer sets with a T7 docking sequence at the 5′ end of both the sense and antisense primers (Supplementary Table 5). Primers were designed as previously described (Ingham et al., 2018). PCR was performed using the following cycles: 98°C for 30s, (98°C 7s, 65°C 10s, 72°C 10s) x35 and 72°C 5 minutes. PCR product was then purified using a Qiagen QIAquick PCR Purification Kit following manufacturers’ instructions. dsRNA was then synthesised using a Megascript® T7 Transcription (Ambion) kit, following manufacturer's instructions (16-hour 37 °C incubation). The dsRNA was cleaned using a MegaClear® Transcription Clear Up (Ambion) kit, with DEPC water, twice heated at 65 °C for 10 min, to elute the sample. The resultant dsRNA product was analysed using a nanodrop spectrometer (Nanodrop Technologies, UK) and subsequently concentrated to 3 μg/μl using a vacuum centrifuge at 35°C. 69nL of dsRNA was subsequently injected into presumed mated, non-blood fed, 3-day old VK7 females immobilised using a CO_2_ block using a NanoInject II. 50 females were injected with each of the transcription factor dsRNA and 50 with dsGFP as a non-endogenous control.

### Insecticide Exposures

25-30 female mosquitoes were exposed to 0.05% deltamethrin impregnated papers for one hour in a standard tube bioassay kit following WHO guidelines. Post-exposure mosquitoes were transferred into holding tubes and maintained on sucrose solution.

### RNA extraction and cDNA synthesis

RNA was extracted from 7-10 female mosquitoes in biological triplicate for each experimental group. RNA was extracted from homogenised mosquitoes using a PicoPure RNA isolation kit (Thermo Fisher, UK) following manufacturers’ instructions and treated with DNAase (Qiagen) to remove any DNA contamination. Quality of RNA was checked using a nanodrop spectrophotometer (Nanodrop Technologies UK). 1-4µg of RNA from each experimental set was reversed transcribed using OligoDTT (Invitrogen) and Superscript III (Invitrogen) according to manufacturers’ instructions. The following experimental groups were used: (i) knockdown efficacy for each transcription factor and the GFP control using females 48-hours post RNAi injection and (ii) pathway validation using females 48-hours after they were exposed to 0.05% deltamethrin for 48-hours post-injection for transcription factors and GFP controls.

### qPCR validation

Quantitative real-time PCR was performed using SYBR Green Supermix III (Applied Biosystems, UK) using an MX3005 and the associated MxPro software v4.10 (Agilent, UK). Primer Blast (NCBI) was used to design primer pairs. Where possible, primers were designed to span an exon junction (Supplementary Table 5). Each 20µl reaction contained 10µl SYBR Green Supermix, 0.3µM of each primer and 1µl of 4ng/µL cDNA. Standard curves for each primer set were used to calculate efficiency, using five 1:5 dilutions of cDNA to ensure that all primer sets met the MIQE guidelines (90-120% efficiency) (Bustin et al., 2009). qPCR was performed with the following conditions: 3 minutes at 95°C, with 40 cycles of 10 seconds at 95°C and 10 seconds at 60°C. Relative expression was normalised against two housekeeping genes: EF (AGAP005128) and S7 (AGAP010592) and analysed using comparative CT method (Schmittgen and Livak, 2008). qPCR was used to determine the efficacy of transcription factor knockdown by comparing cDNA taken from mosquitoes 48-hours post dsRNA injection for each transcription factor and comparing it to GFP-injected controls all taken from the same mosquito generation. To validate findings in the network, qPCR was performed on dsRNA injected mosquitoes exposed to 0.05% deltamethrin at 48-hours post injection, these mosquitoes were then left for a further 48-hours before harvesting; again, transcription factor injected mosquitoes were compared to the dsGFP injected controls.

## Author Contributions

VAI and FD designed and implemented the experiment. SCN performed the SILGGM analysis, FD modified and implemented the dynamic Bayesian network, SE provided rearing, bioassay and molecular biology support, VAI performed the lab-based experiments and analysed all data. VAI and FD drafted the manuscript.

## Data Availability

The datasets used in this experiment are available at ArrayExpress under E-MTAB-9422 and E-MTAB-9423. The authors declare that all other data supporting the findings of this study, are available within the article and its Supplementary Information files or are available from the authors upon request.

## Code Availability

Code used for analysis in this study is available on GitHub. Network visualisation is available at https://github.com/VictoriaIngham/NetworkVis_TimeCourse, model code is available on the CRAN repository: https://cran.r-project.org/web/packages/EDISON and full analysis is available at https://github.com/FrankD/AnophelesInsecticideExposure.

## Declaration of Competing Interest

The authors declare that they have no known competing financial interests or personal relationships that could have appeared to influence the work reported in this paper.

## Appendix Supplementary materials

Image, application 1

Image, application 2

Image, application 3

Image, application 4

Image, application 5

Image, application 6

Image, application 7

Image, application 8

Image, application 8

Image, application 8

Image, application 8

Image, application 8

Image, application 8
