# Supplementary material for: Capturing the transcription factor interactome in response to sub-lethal insecticide exposure
Source: Curr Res Insect Sci. 2021 Jul 25;1:100018. doi: 10.1016/j.cris.2021.100018 (PMC8702396; doi:10.1016/j.cris.2021.100018)
Supplement: Supplementary file 1 [file mmc1.pdf]

Appendix: Network inference using a dynamic Bayesian model

Victoria A Ingham, Sara Elg, Sanjay C Nagi and Frank Dondelinger

1 Notation

Let $V = \{1 \dots p\}$ index a set of variables that have been measured in molecular assays and $X_{j,t}$ be a random variable corresponding to the measurement of variable $j \in V$ at time point t. The data are observed at a set of discrete time points, hence we write $t \in \{1 \dots T\}$ where T is the total number of time points.

We will use $X_t \in \mathbb{R}^p$ to denote the complete observed vector at time t and $X = (X_1 \dots X_T)^{\mathrm{T}}$ to denote the complete $T \times p$ data matrix. For a subset $A \subseteq V$ of variable indices, we use $X_{A,t}$ to denote the random vector formed by selecting the corresponding elements of X_t.

Let G denote a graph-valued model parameter and Θ any other parameters needed to specify the model (e.g. regression coefficients, reaction rates etc.). Then, we consider likelihoods of the form $p(X_1 \dots X_T \mid G, \Theta)$. We denote the edge set of a graph G as $E(G)$, or simply E when the graph of interest is clear from context.

We will generically use $\mathcal{G}$ to denote the space of permissible graphs. Additionally, we use $\mathrm{N}(\cdot \mid \mu, \Sigma)$ to denote a multivariate normal density with mean μ and covariance matrix Σ, I to denote the identity matrix and $\mathbb{I}(\cdot)$ to denote the indicator function.

2 Dynamic Bayesian networks

Bayesian networks (BNs) are graphical models (Koller and Friedman, 2009) based on directed acyclic graphs (DAGs). In a BN the likelihood factors into terms in which each variable is conditioned only on its parents in the DAG G, i.e.

$$p(X \mid G, \Theta) = \prod_{j=1}^{p} p(X_j \mid X_{\mathrm{Pa}_G(j)}, \theta_j) \tag{1}$$

where $\mathrm{Pa}_G(j) = \{i \in V : (i,j) \in E(G)\}$ is the set of parents of node j in the graph G and $\Theta = (\theta_1 \ldots \theta_p)$ are parameters required to fully specify the conditional distributions.

In a dynamic Bayesian network (DBN) the model has an explicit discrete time index. The model can be formulated as a BN whose DAG has one vertex for each variable at each discrete time point, i.e. with $p \times T$ vertices in total (Murphy, 2002; Husmeier, 2003; Hill et al., 2012). We denote this graph by G_{full}. We assume that the edges are only one step forward in time (i.e. the model is first order Markov) and that neither the dependence pattern nor parameters change with time in the sense that any edge between variables $i \in V$ and $j \in V$ appears between all pairs of successive time points (or none) and the required model parameters are also unchanging. This type of DBN is often referred to as "feedforward" to reflect the graph structure and stationary to reflect the unchanging nature of the model.

The network structure of such a model does not change over time and the DAG G_{full} is a redundant representation since each edge is repeated between each pair of successive times. A more compact representation is as a bipartite directed graph $G_{\mathrm{bipartite}}$ with only two time slices each with p nodes, with the two slices understood to represent successive times $(t-1, t)$. A still more compact representation is as a p-node directed graph G_{reduced} in which all edges are understood to go forwards in time between successive time points. Due to the fact that DAG G_{full} can be obtained by "unrolling" G_{reduced} through time, it is often referred to as the "unrolled" representation. These three representations are shown in Figure 1 for an illustrative example.

Although G_{full} must be acyclic to obtain a valid overall BN model, the reduced graph G_{reduced} may have cycles. This is due to the fact that its edges are understood to go forward in time, hence the full graph G_{full} corresponding to an arbitrary directed G_{reduced} is always acyclic. For notational simplicity in the remainder of this section we use the reduced graph and denote it by G $(= G_{\mathrm{reduced}})$.

Using the reduced graph G the likelihood for a stationary feedforward DBN can be written as

$$p(X \mid G, \Theta) = \prod_{j=1}^{p} p(X_{j,1} \mid \theta_j^{(0)}) \prod_{t=2}^{T} p(X_{j,t} \mid X_{\mathrm{Pa}_G(j),t-1}, \theta_j), \tag{2}$$

where as above, θ_j are parameters governing the conditional distributions, $\theta_j^{(0)}$ are parameters governing the (marginal) distribution at the first time point and $\Theta = (\theta_j^{(0)}, \theta_j)_{j=1\ldots p}$. Note that due to the stationary nature of the model, the parameters do not have a time index.

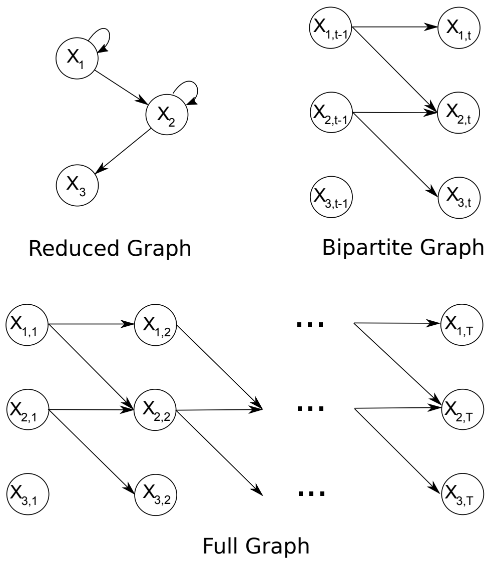

Figure 1: Different graphical representations for a DBN. The top left shows the reduced graph G_{reduced}, where the temporal structure is implicit. The top right shows the bipartite graph $G_{\text{bipartite}}$ between any two time slices $t-1$ and t. The bottom shows the full (or "unrolled") graph G_{full} over all time slices.

Thus, in our model each variable depends on a subset of variables at the previous time point, with the subset given by the parent set of the variable in the graph G. However, we additionally allow for dependence on the variables correcting for ageing and circadian rhythms, which are observed at the same time point as the dependent variables (see main text).

3 Model formulation

For stationary feedforward DBNs the likelihood Eq. (2) is expressed in terms of the reduced graph G ($= G_{\text{reduced}}$; see above) and it is this graph that we treat as the estimand. A common way to proceed is via a Bayesian formulation. Since the main object of interest is G, we consider the posterior probability distribution over candidate graphs. This can be written as

$$P(G \mid X) \quad = \quad \frac{p(X \mid G)\, P(G)}{\sum_{G \in \mathcal{G}} p(X \mid G)\, P(G)} \tag{3}$$

where $\mathcal{G}$ is the space of all directed graphs (with p vertices; not necessarily acyclic) and $P(G)$ is the prior on the graph space. Note that the graph

space is not restricted to acyclic graphs due to the fact that it is the reduced graph that is the estimand (see above).

We select a truncated Poisson prior with mean Λ and maximum $\bar{s}$ for the number of parents s_j for target gene j: $P(s_j^h|\Lambda) \propto \frac{\Lambda^{s_j}}{s_j!}\mathbb{I}_{\{s_j \leq \bar{s}\}}$. Conditional on s_j, the prior for the parent set G_j of target gene j is a uniform distribution over all parent sets with cardinality s_j: $P(G_j \mid |G_j| = s_j) = 1/\binom{p}{s_j}$. The overall prior over the parent sets is given by marginalization:

$$P(G_j|\Lambda) = \sum\nolimits_{s_j=1}^{\bar{s}} P(G_j|s_j)P(s_j|\Lambda). \tag{4}$$

We can derive the posterior probability that a specific edge (i,j) is included in the model as

$$P((i,j) \in E(G) \mid X) = \sum_{G \in \mathcal{G}} \mathbb{I}[(i,j) \in E(G)]\, P(G \mid X). \tag{5}$$

The term $p(X \mid G)$ is known as the marginal likelihood since it is obtained by marginalizing over model parameters as

$$\begin{aligned} p(X \mid G) &= \int p(X \mid G, \Theta)\, p(\Theta)\, \mathrm{d}\Theta & (6)\\ &= \prod_{t=2}^{T}\prod_{j=1}^{p} \int p(X_{j,t} \mid X_{\mathrm{Pa}_G(j),t-1}, \theta_j)\, p(\theta_j)\, \mathrm{d}\theta_j & (7) \end{aligned}$$

where we have used (2) and $p(\Theta)$ is a prior on model parameters, assumed to factor as $p(\Theta) = \prod_j p(\theta_j)$.

Under conjugate formulations the quantity

$$\int p(X_{j,t} \mid X_{\mathrm{Pa}_G(j),t-1}, \theta_j)\, p(\theta_j)\, \mathrm{d}\theta_j$$

can be obtained in closed form. We choose a normal linear model, i.e.

$$X_{j,t} \mid X_{\mathrm{Pa}_G(j),t-1}, \theta_j \sim \mathrm{N}(X_{\mathrm{Pa}_G(j),t-1}\beta_j, \sigma_j^2), \tag{8}$$

where $\theta_j = (\beta_j, \sigma_j^2)$, β_j is a p-vector of model coefficients and σ_j^2 is a noise variance.

Using a conjugate prior (here, normal-inverse-gamma) then allows the marginal likelihood to be obtained in closed form (see Lèbre et al., 2010, and references therein for details).

However, even with a closed-form marginal likelihood, characterizing the posterior over G is complicated by the size of the space $\mathcal{G}$, which absent any further constraints has cardinality 2^{p^2}.

4 Inference using MCMC

Markov chain Monte Carlo (MCMC) methods are widely used to sample from the posterior $P(G \mid X)$. These methods work by constructing a Markov chain (whose states are in this case graphs in the space $\mathcal{G}$), whose stationary distribution is the desired posterior. A common approach is to use a Metropolis-Hastings sampler with small changes to the graph (e.g. single edge changes) used to form the proposal distribution (this is the mechanism by which the sampler explores the graph space). Such samplers are asymptotically valid but can be very slow to converge for large graph spaces. Efficient samplers for (D)BNs remain an active area of research (Grzegorczyk and Husmeier, 2008; Goudie and Mukherjee, 2016)

In the special case of feedforward DBNs posterior inference is greatly simplified by the structure of the model. In particular, posterior edge probabilities can be computed via a variable selection approach in which each node is treated separately. An additional assumption of in-degree bounded by $\hat{s} = 3$ then reduces the problem to polynomial in p.

This means that fully Bayesian analysis of feedforward DBNs with conjugate priors is in fact feasible for very large problems, with p in the hundreds or thousands, and can be parallelized over target genes. In this work we use the MCMC algorithm described in more detail in Lèbre et al., 2010; Dondelinger, Lèbre, and Husmeier, 2012.

References

Dondelinger, F., S. Lèbre, and D. Husmeier (2012). "Non-homogeneous dynamic Bayesian networks with Bayesian regularization for inferring gene regulatory networks with gradually time-varying structure". *Machine Learning.*

Goudie, R. J. and S. Mukherjee (2016). "A Gibbs sampler for learning DAGs". *The Journal of Machine Learning Research* 17.1, pp. 1032–1070.

Grzegorczyk, M. and D. Husmeier (2008). "Improving the structure MCMC sampler for Bayesian networks by introducing a new edge reversal move". *Machine Learning* 71.2, pp. 265–305.

Hill, S. M. et al. (2012). "Bayesian inference of signaling network topology in a cancer cell line". *Bioinformatics* 28.21, pp. 2804–2810.

Husmeier, D. (2003). "Sensitivity and specificity of inferring genetic regulatory interactions from microarray experiments with dynamic Bayesian networks". *Bioinformatics* 19.17, pp. 2271–2282.

Koller, D. and N. Friedman (2009). *Probabilistic graphical models: principles and techniques*. MIT press.

Lèbre, S. et al. (2010). "Statistical inference of the time-varying structure of gene-regulation networks". *BMC Systems Biology* 4.130.

Murphy, K. P. (2002). "Dynamic Bayesian networks: Representation, inference and learning". dissertation. University of California, Berkeley.
