# Supplementary material for: Capturing the transcription factor interactome in response to sub-lethal insecticide exposure
Source: Curr Res Insect Sci. 2021 Jul 25;1:100018. doi: 10.1016/j.cris.2021.100018 (PMC8702396; doi:10.1016/j.cris.2021.100018)
Supplement: Supplementary file 2 [file mmc2.docx]

Appendix: Simulation Study for Validation Experiments

Victoria A Ingham, Sara Elg, Sanjay C Nagi and Frank Dondelinger

6th June 2021

## Motivation

We experimentally validated the gene-transcription factor associations detected using the dynamic Bayesian network analysis. Due to resource constraints, it is only possible to validate a small subset of the detected associations. This raises the question of the optimal number of target genes and associations per target gene to investigate to obtain a reasonable estimate of the precision of our detected associations. As we do not have an external validation set, the only way to determine this is via a validation study.

## Simulation Setup

We assume that the mean number of genes regulated by each TF $\mu_{reg}=10$, and the actual number of regulated genes $n_{reg,j}$ for transcription factor $j$ follows a Poisson distribution with mean $\mu_{reg}$. We simulate the true associations directly using a Bernoulli process with parameter $p=n_{reg,j}/n_{targets}$ where $n_{targets}$ is the number of possible target genes for each transcription factor.

To simulate the predicted associations, we set the rate of true positives (correctly predicted associations) to be 0.75, and the rate of true negatives (correctly predicted non-associations) to be 0.997. This results in an average precision of ~0.6 and an average recall of ~0.72, which seem like reasonably conservative values. Note that because the set of associations is small, and our network model is sparse, we would expect it to be easier to detect a non-association than an association.

We assume that transcription factors and regulated genes to test are selected randomly, and that the qPCR knockdown test is 100% accurate. We simulate 1000 different populations of predicted and true associations for each number of investigated target genes and transcription factors, and compare the estimate of the precision when using the a subset of the transcription factors and genes to the actual precision in our simulated setup.

## Results


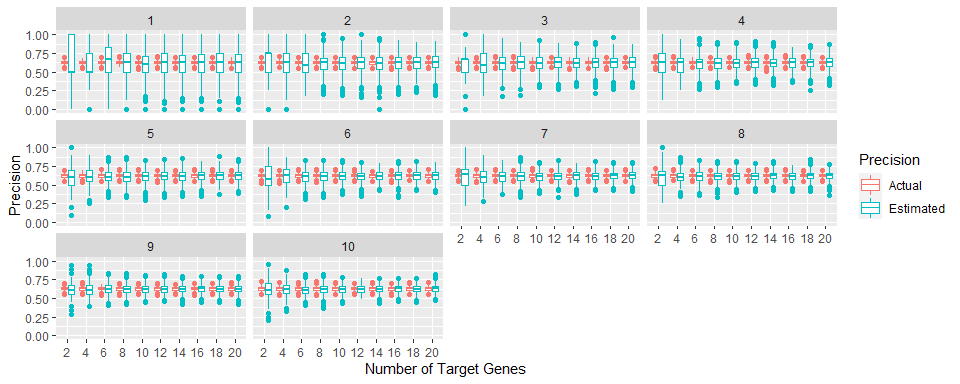


Actual and estimated precision for increasing number of transcription factors and target genes. Each plot is labeled with the number of transcription factors tested.

We observe that the variance of the estimate of the precision decreases as we test more associations. Figure 1 shows the effect of increasing the number of transcription factors (subsequent plots) and target genes (x-axis within each). This leads us to conclude that in order to get a reliable estimate of the precision, we need at least 4-7 transcription factors with 4 target genes tested for each of them. Figure 2 shows that testing 4 regulatory relationships for 7 transcription factors has a 70% chance of obtaining an estimate of the precision that falls within 10% of the true precision, and a 97% chance of obtaining an estimate that falls within 20% of the true precision. For 4 transcription factors, the chance of obtaining an estimate within 10% of the true precision falls to 56%, and the chance of obtaining an estimate within 20% is 89%.


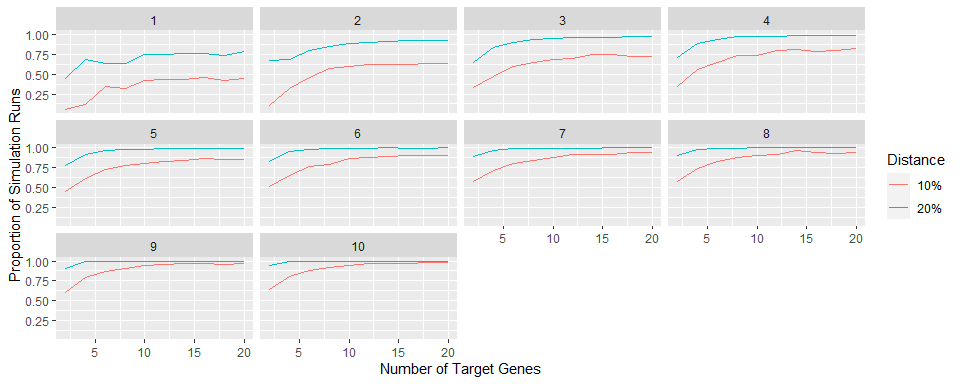


Proportion of simulation runs where the precision estimate falls within the given distance (10% or 20%) of the true preision, for increasing number of transcription factors and target genes. Each plot is labeled with the number of transcription factors tested.
