# Supplementary figures and images for: Capturing the transcription factor interactome in response to sub-lethal insecticide exposure
Source: Curr Res Insect Sci. 2021 Jul 25;1:100018. doi: 10.1016/j.cris.2021.100018 (PMC8702396; doi:10.1016/j.cris.2021.100018)

# Knockdown post-exposure

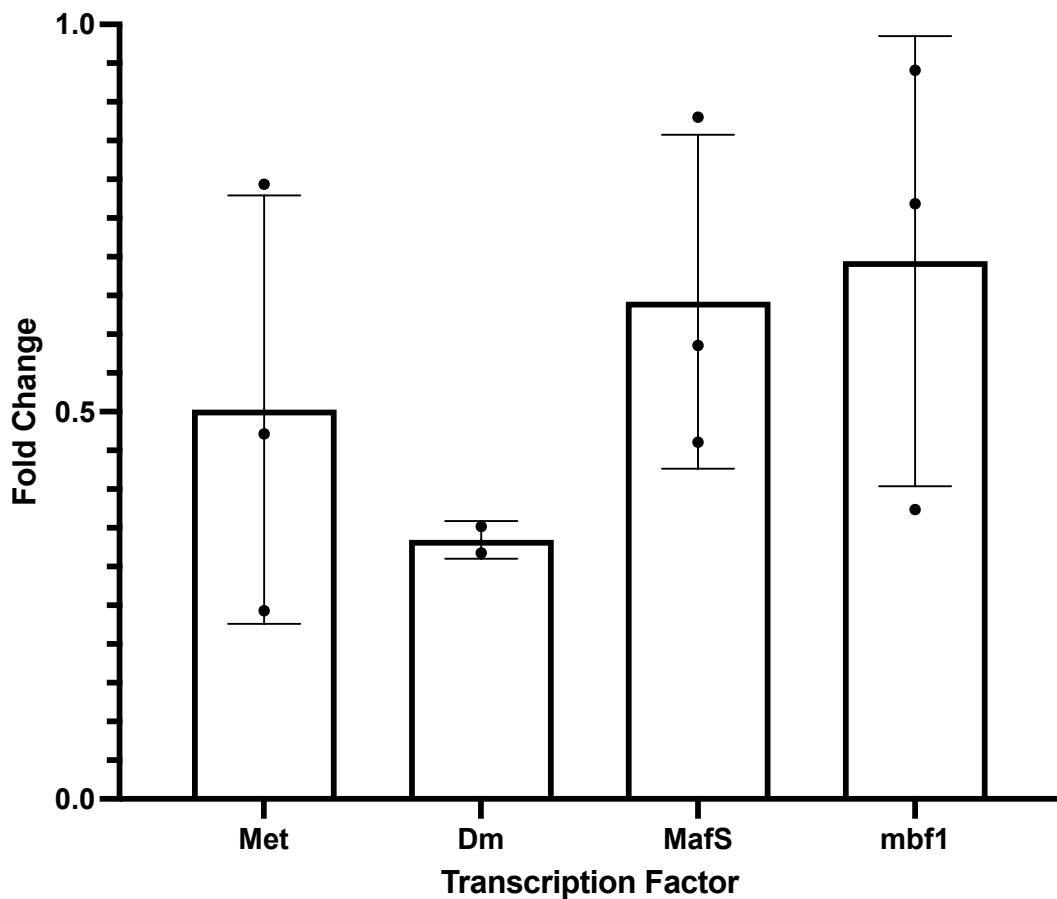

Supplement: Supplementary file 3 [file mmc3.pdf]

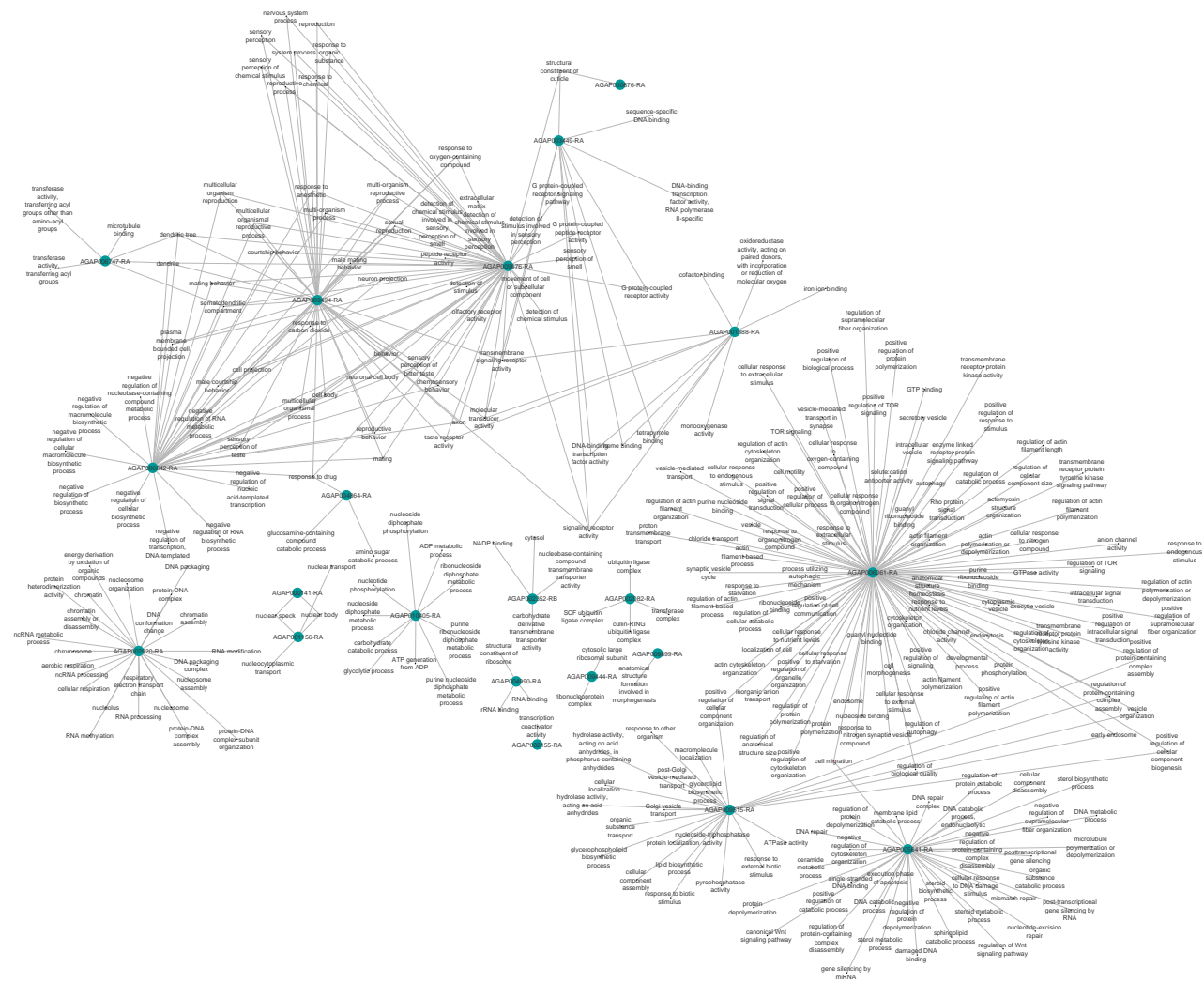

Supplement: Supplementary file 4 [file mmc4.pdf]

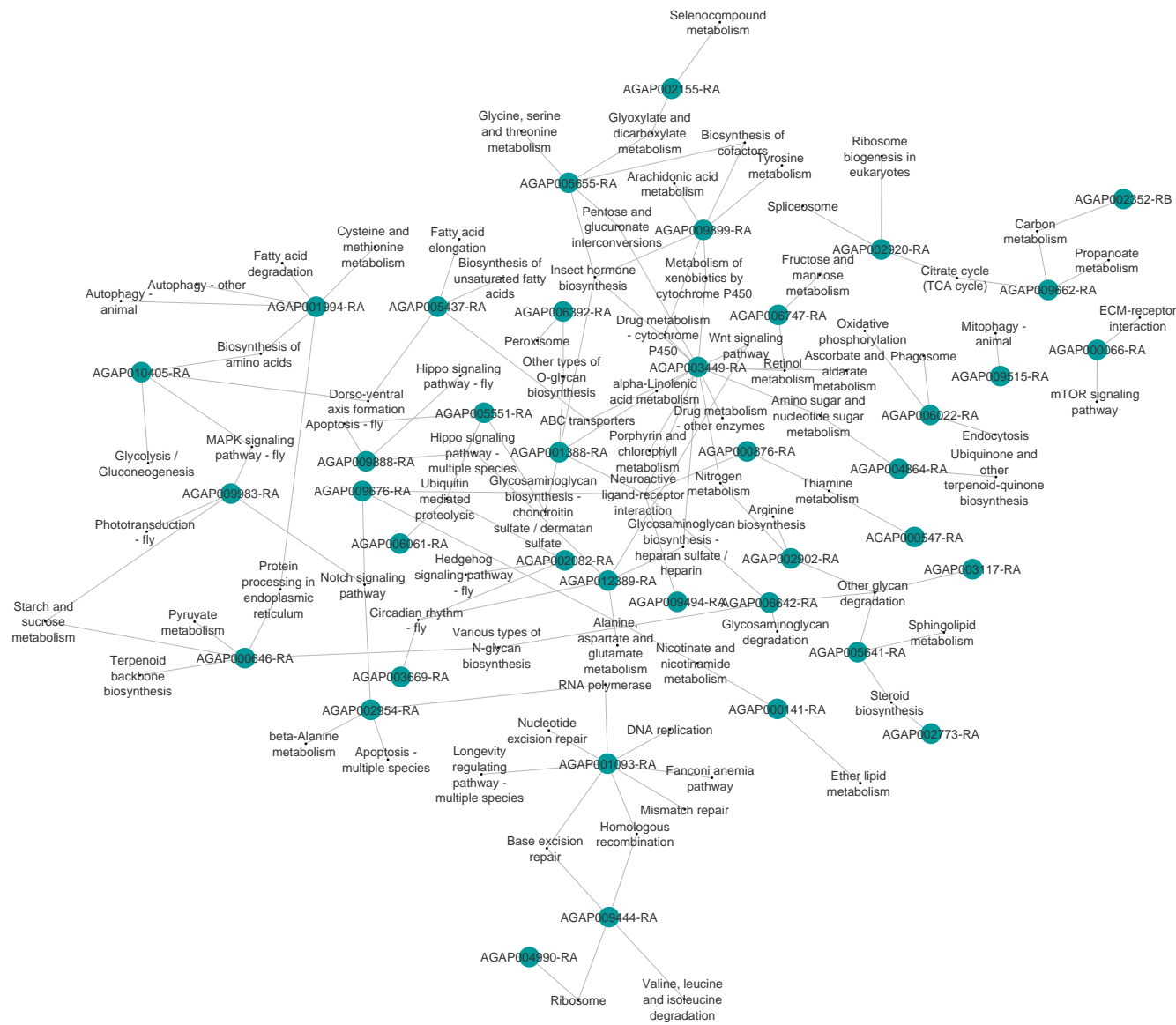

Supplement: Supplementary file 5 [file mmc5.pdf]

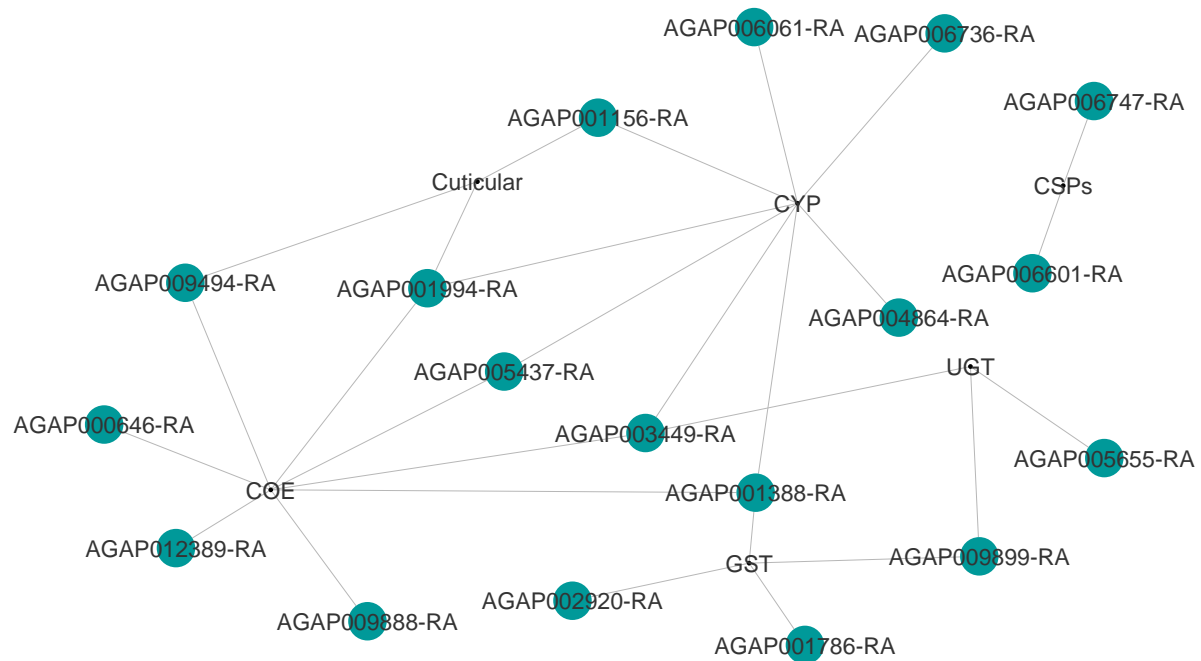

Supplement: Supplementary file 6 [file mmc6.pdf]

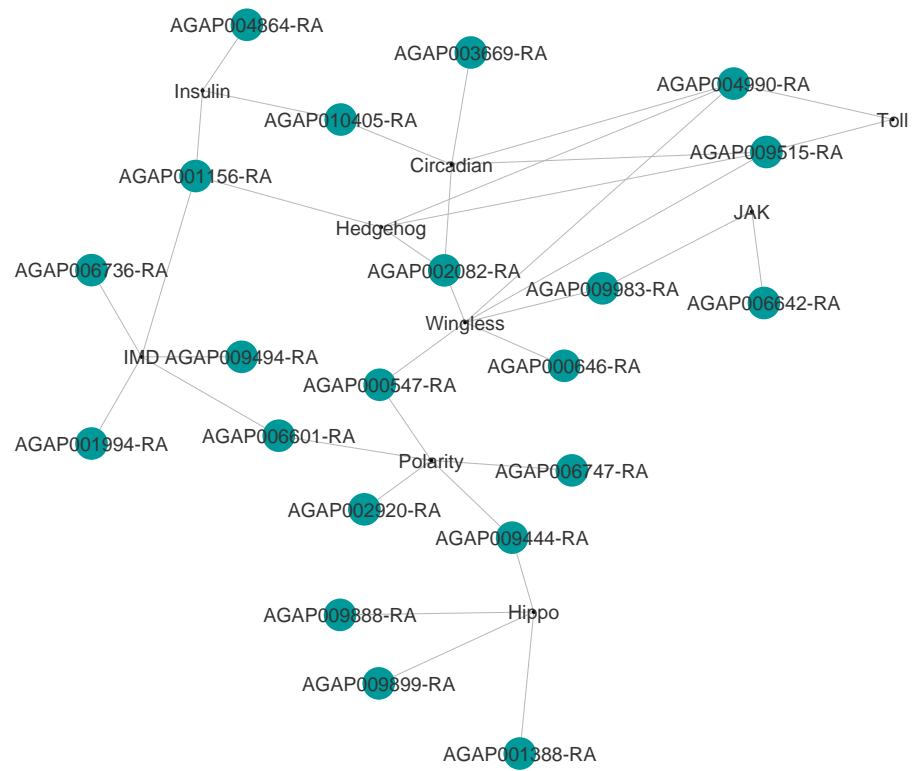

Supplement: Supplementary file 7 [file mmc7.pdf]
