# Supplementary material for: Capturing the transcription factor interactome in response to sub-lethal insecticide exposure
Source: Curr Res Insect Sci. 2021 Jul 25;1:100018. doi: 10.1016/j.cris.2021.100018 (PMC8702396; doi:10.1016/j.cris.2021.100018)

**Supplementary Figure 1: Transcription factor knockdown.** qPCR of relative fold change of transcription factor dsRNA injections compared to dsGFP injected control 48 hours post-exposure.

**Supplementary Figure 2: Visualisation of GO term enrichments.** Significant (p < 0.05) GO terms for each transcription factor (blue). Thickness of the edge represents significance, with thicker edges having smaller p values.

**Supplementary Figure 3: Visualisation of KEGG term enrichments.** Significant (p < 0.05) KEGG terms for each transcription factor (blue). Thickness of the edge represents significance, with thicker edges having smaller p values.

**Supplementary Figure 4: Visualisation of detoxification families.** Significant (p < 0.05) detoxification families for each transcription factor (blue). Thickness of the edge represents significance, with thicker edges having smaller p values.

**Supplementary Figure 5: Visualisation of Reactome enrichments.** Significant (p < 0.05) Reactome terms for each transcription factor (blue). Thickness of the edge represents significance, with thicker edges having smaller p values.

**Supplementary Figure 6: Estimating the impact of transcription factor choice.** Edges were split into strong (those that had marginal posterior probability >0.75 in the original analysis), and weak (all other edges). Weak edges mostly agree across the two estimations, while there is a slight bias in the strong edges; the marginal posterior probability tends to be slightly lower when 25% of the transcription factors have been changed.


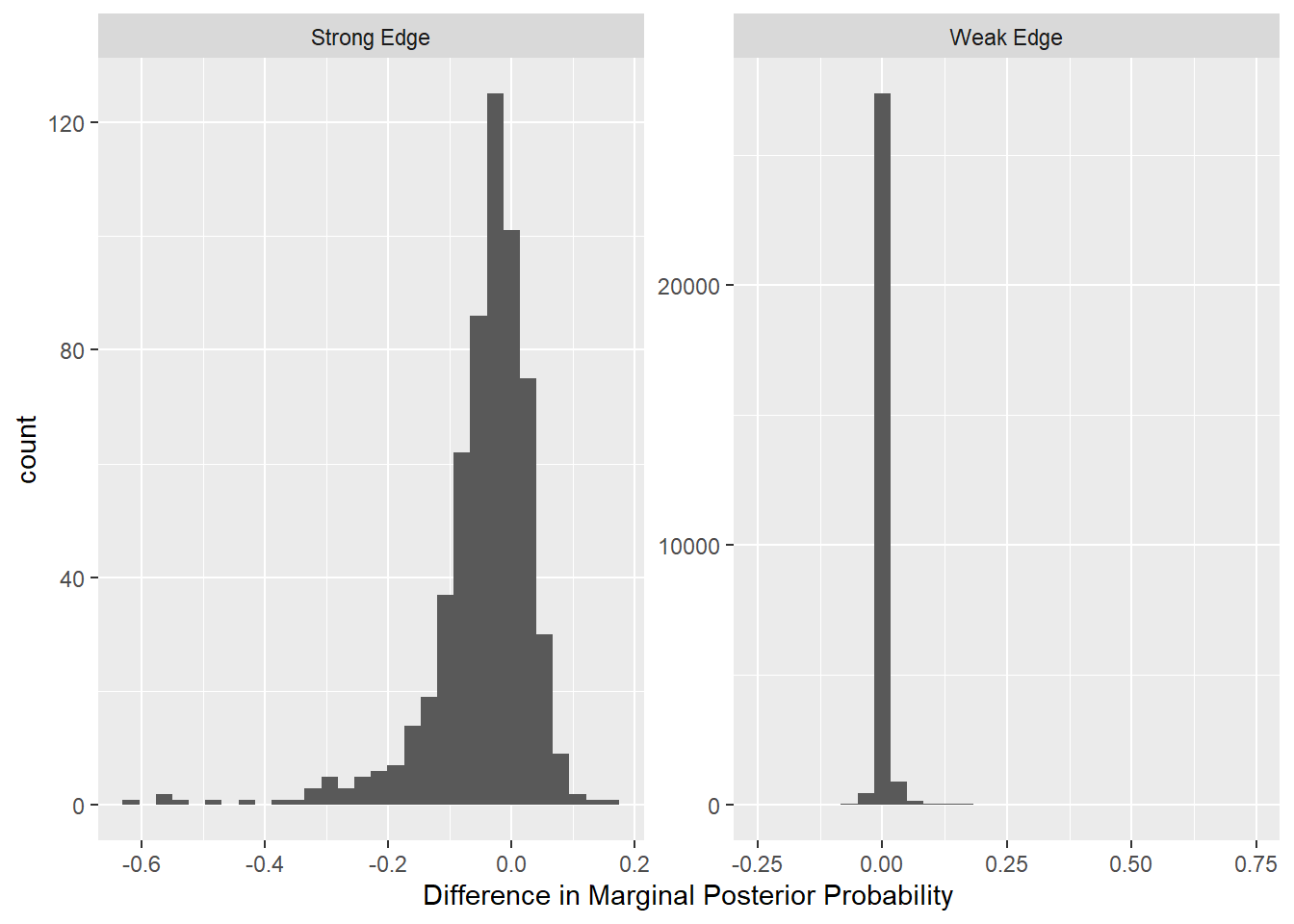


**Supplementary Figure 7: Estimating the computational cost of increasing the number of transcription** **factors.** To estimate the upper limit of transcription factors it was feasible to run in this analysis, the time for ten randomly selected target genes and p transcription factors, where p∈(5,100) is shown. Given the 14911 target genes, 44 transcription factors takes around 6-days to run.


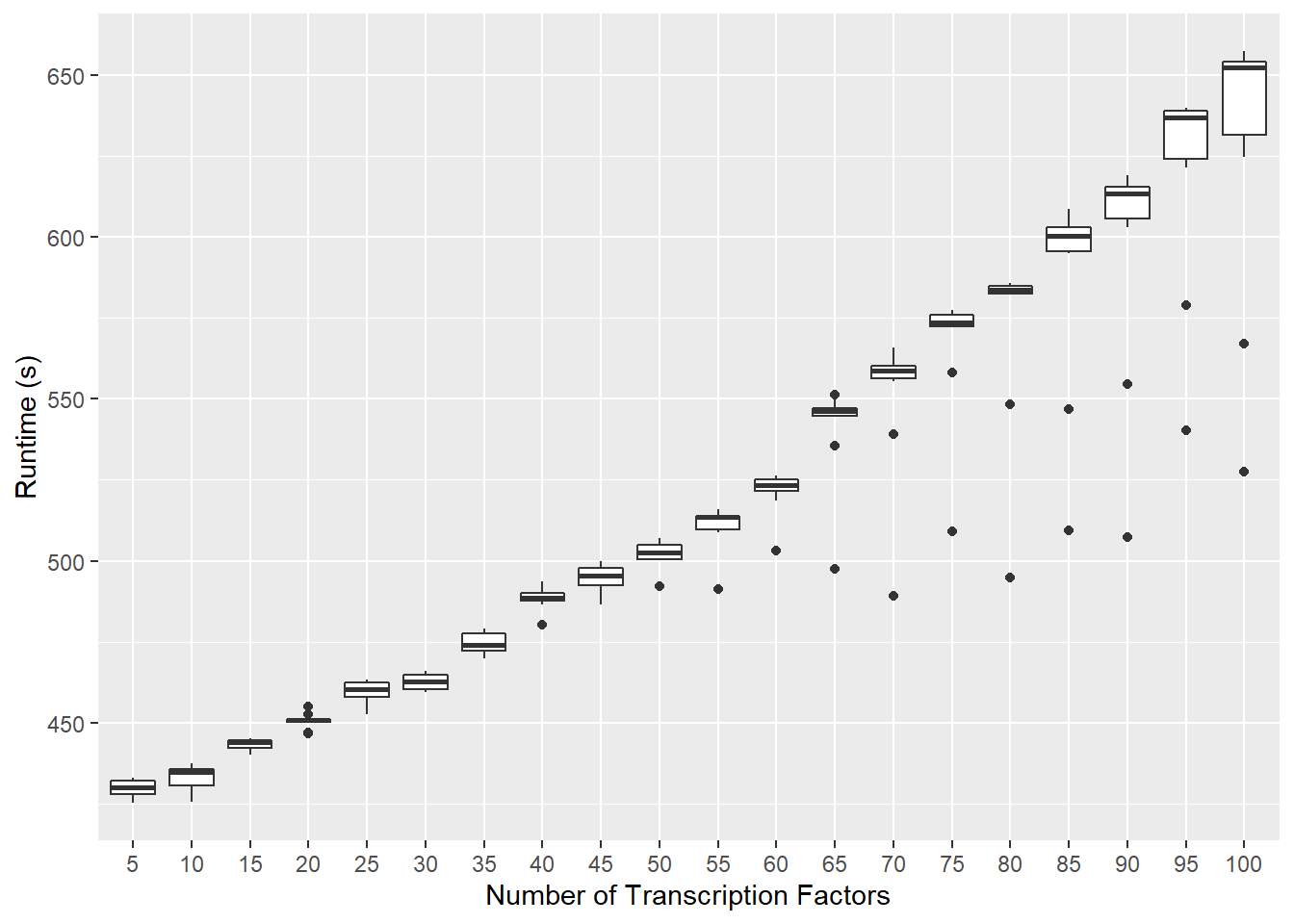

Supplement: Supplementary file 8 [file mmc8.docx]
